# Surface Texturing-Plasma Nitriding Duplex Treatment for Improving Tribological Performance of AISI 316 Stainless Steel

**DOI:** 10.3390/ma9110875

**Published:** 2016-10-27

**Authors:** Naiming Lin, Qiang Liu, Jiaojuan Zou, Junwen Guo, Dali Li, Shuo Yuan, Yong Ma, Zhenxia Wang, Zhihua Wang, Bin Tang

**Affiliations:** 1 Research Institute of Surface Engineering, Taiyuan University of Technology, Taiyuan 030024, Shanxi, China; liuqiang0116@tyut.edu.cn (Q.L.); zoujiaojuan0025@tyut.edu.cn (J.Z.); guojunwen0081@tyut.edu.cn (J.G.); lidali0197@tyut.edu.cn (D.L.); yuanshuo0101@lingk. tyut.edu.cn (S.Y.); mayong@tyut.edu.cn (Y.M.); wangzhenxia@tyut.edu.cn (Z.W.); tangbin@tyut.edu.cn (B.T.); 2 Shanxi Key Laboratory of Material Strength and Structure Impact, Taiyuan University of Technology, Taiyuan 030024, Shanxi, China; wangzhihua@tyut.edu.cn; 3 Department of Chemical and Materials Engineering, University of Alberta, Edmonton, AB T6G 1H9, Canada

**Keywords:** surface texturing, plasma nitriding, duplex treatment, tribological performance, austenitic stainless steel

## Abstract

Surface texturing-plasma nitriding duplex treatment was conducted on AISI 316 stainless steel to improve its tribological performance. Tribological behaviors of ground 316 substrates, plasma-nitrided 316 (PN-316), surface-textured 316 (ST-316), and duplex-treated 316 (DT-316) in air and under grease lubrication were investigated using a pin-on-disc rotary tribometer against counterparts of high carbon chromium bearing steel GCr15 and silicon nitride Si_3_N_4_ balls. The variations in friction coefficient, mass loss, and worn trace morphology of the tested samples were systemically investigated and analyzed. The results showed that a textured surface was formed on 316 after electrochemical processing in a 15 wt % NaCl solution. Grooves and dimples were found on the textured surface. As plasma nitriding was conducted on a 316 substrate and ST-316, continuous and uniform nitriding layers were successfully fabricated on the surfaces of the 316 substrate and ST-316. Both of the obtained nitriding layers presented thickness values of more than 30 μm. The nitriding layers were composed of iron nitrides and chromium nitride. The 316 substrate and ST-316 received improved surface hardness after plasma nitriding. When the tribological tests were carried out under dry sliding and grease lubrication conditions, the tested samples showed different tribological behaviors. As expected, the DT-316 samples revealed the most promising tribological properties, reflected by the lowest mass loss and worn morphologies. The DT-316 received the slightest damage, and its excellent tribological performance was attributed to the following aspects: firstly, the nitriding layer had high surface hardness; secondly, the surface texture was able to capture wear debris, store up grease, and then provide continuous lubrication.

## 1. Introduction

Material scientists and engineers have long been committed to designing and producing new materials that are both wear-resistant (hard or friction-reduction) and corrosion-resistant (mechanical isolation, chemical stability, or passivation) and that can meet the increasing challenges and demands over a wide range of modern industrial applications in aggressive and harsh conditions. In these situations, another way of achieving improvement in performance is utilizing surface modification technologies involving coating formulations on the surfaces of existing materials and obtained expected properties [1]. Surface modification technologies allow the realization of a favorable compromise between cost and performance by endowing the material surfaces with a high hardness value, effective friction-reduction, excellent corrosion resistance, and promising mechanical performance, without affecting the entire structure of the material [2]. Meanwhile, coatings/films/layers deposited on the surfaces of different substrates via surface modification technologies can increase the operating life and widen the applying field [3].

Thanks to the excellent general corrosion resistance, nonmagnetic properties, acceptable biocompatibility, promising mechanical properties, and desirable formability and weldability, austenitic stainless steels (ASS‘s) have been confirmed as the most widely used family of stainless steels in the field, ranging from civilian goods to military equipment [4,5,6,7,8,9,10,11,12]. Engineering components used in severe environments demand more technical and physical reliability to guarantee safe operations and longer service life [2]. Generally, ASS‘s are extensively used in structural applications where corrosion resistance is a crucial requirement; however, ASS‘s components might fail during service due to wear rather than corrosion-related degradation problems in engineering practice [2,5]. ASS‘s are notorious for their poor tribological characteristics, such as low sliding wear resistance, high friction, and the formation of strong adhesion [5,6,7]. As a result, ASS‘s often suffer surface damages when they slide against themselves or other metals, which has limited their tribological applications [13]. For these purposes, surface modification technologies, e.g., thermochemical diffusion treatments (such as nitriding, carburizing, and nitrocarburizing), surface alloying, spraying, laser cladding, physical vapor deposition (PVD), surface mechanical attrition treatment (SMAT), and friction stir processing have been conducted to enhance the corrosion resistance and tribological property of ASS‘s by forming coatings, films, and layers on the surfaces [14,15,16,17,18,19,20,21].

**Figure 1:**
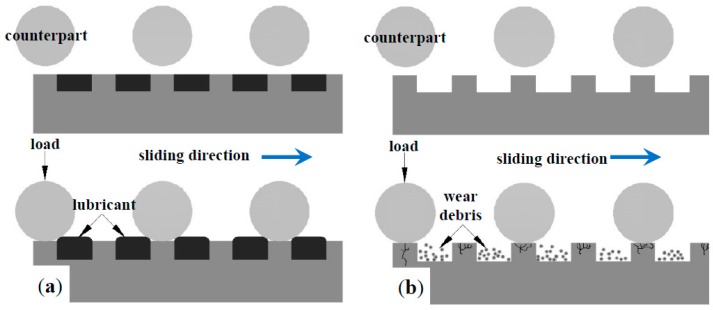
Function diagram of surface texturing: (**a**) storing lubricants; (**b**) capturing wear debris.

**Figure 2:**
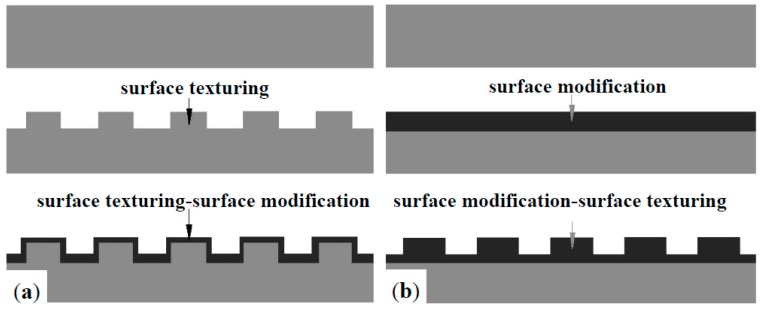
Diagram of surface texturing-based surface duplex treatments: (**a**) surface texturing-surface modification; (**b**) surface modification-surface texturing.

Apart from the surface modification technologies mentioned above, proper design on surface morphology can also play positive rules in the tribological performance of ASS‘s according to recent bionic achievements [22]. Design on surface morphology usually aims to obtain a regular pattern on the surface, which is inspired by rough surfaces in the natural world [23,24,25]. The received artificial surface patterns with typical distributing characteristics such as dimple, groove and protrusion were collectively named as surface texturing [26,27,28]. In recent decades, surface texturing has been considered an effective method to improve the tribological performance of mechanical parts with great success [29]. Generally, the active roles of surface texturing in tribological performance lie in two main aspects (see Figure 1): firstly, surface texturing can store up the grease and thus offer sustainable lubrication; secondly, it can capture debris generated during service and thus minimize abrasive wear [30,31]. Since the advantages of surface modification technologies and surface texturing improve the tribological performance of materials, some surface modification-surface texturing duplex treatments have been conducted. One kind is the “surface texturing-surface modification”, and the other kind is the “surface modification-surface texturing”, as shown in Figure 2 [32,33,34,35,36,37,38,39,40,41,42,43,44,45]. A database has been created, and reference information for practical applications has been provided.

Our group concentrated on improving the tribological performance of AISI 316 stainless steel (hereafter referred to as 316) by surface texturing-plasma nitriding duplex treatment. It is widely reported that ASS‘s have a poor localized pitting corrosion-resistant ability in a chloride ion-rich environment, the destructive surface of ASS‘s with numerous pits or dimples is a marked characteristic of pitting corrosion [10,46,47]. Plasma nitriding treatment, which is one of the most extensively used surface treatment technologies that are available for steels and ASS‘s to obtain improved wear and/or corrosion resistance and fatigue strength, has been under development for several decades [14,48,49,50,51]. In light of the background above, we firstly obtained a groove-like surface texture on 316 by electrochemical processing in a 15 wt % solution of sodium chloride, and the surface-textured 316 was then treated with plasma nitriding. The sliding tribological performance of 316, plasma-nitrided 316 (PN-316), surface-textured 316 (ST-316), and duplex-treated 316 (DT-316) in air and under grease lubrication against counterparts of high carbon chromium bearing steel GCr15 and silicon nitride Si_3_N_4_ balls were comparatively investigated.

## 2. Materials and Methods

The specimens used in this work were prepared at a size of 25 mm × 3 mm with an electro-spark wire-electrode cutting machine from a cold-drawn 316 rod. The chemical compositions (wt %) of the 316 are S 0.001; P 0.020; N 0,024; C 0.031; Si 0.45; Ti 0.211; Cu 0.31; Mn 1.66; Mo 2.10; Cr 16.85; Ni 11.75; and Fe balance. All raw samples were finely ground using SiC abrasive papers down to 800# followed by ultrasonic cleaning in acetone bath.

Open circuit potential (OCP) and potentiodynamic polarization tests are usually conducted in a sodium chloride solution to estimate the corrosion resistance of materials by employ of an electrochemical measurement system [52]. In this work, OCP and potentiodynamic polarization tests were performed on the 316 samples in a 15 wt % NaCl solution to obtain a groove-like surface texture. The corrosion cell, which contained 1000 mL of electrolyte, was combined with a typical three-electrode configuration. A saturated calomel electrode (SCE) was used as the reference electrode and a platinum plate was used as the counter electrode (CE). The ground 316 specimens were employed as working electrodes (WEs). OCP measurements immediately began after the samples were immersed into the 15 wt % NaCl solution. The potentiodynamic polarization experiments were started after the 900 s immersion of the samples in the test solution. The potentiodynamic polarizations were swept from −250 to 2000 mV vs. OCP scanned upwards at a rate of 1 mV/s. As this work concentrated on the tribological behaviors of 316 and treated 316; therefore, the electrochemical corrosion behaviors of each tested 316 was not described and discussed here. Plasma nitriding was employed to treat ground 316 and ST-316 according to the literature [53,54].

A scanning electron microscope (SEM) equipped with energy dispersive X-ray spectroscopy (EDS) was applied to observe the surface morphological images and characterize surface elementary compositions of ground 316, ST-316, PN-316, and DT-316 samples. The phase constitutions of PN-316 and DT-316 were identified via X-ray diffraction (XRD). The cross-sectional morphologies of PN-316 and DT-316 were also observed via SEM. A microhardness tester was employed to measure the surface hardness of the 316, ST-316, PN-316, and DT-316 samples using a Vickers indenter under a load of 100 g for a dwell time of 20 s. For comparative purposes, all the friction and wear tests were carried out on a rotary wear testing machine with the following identical parameters: disc wearing against counter balls at 303 K with a gyration radius of 6 mm, a normal load of 30 N, and a sliding velocity of 300 r/min for 1 h. High carbon chromium bearing steel balls (commonly referred as GCr15 in China with a hardness of about 700 HV, the nominal composition-wt % of GCr15 contains C: 0.95–1.05, Mn: 0.25–0.45, Si: 0.15–0.35, S: ≤0.025, P: ≤0.025, Cr: 1.40–1.65, Mo: ≤0.10, Ni: ≤0.30, Cu: ≤0.25, and Fe balance) and silicon nitride (Si_3_N_4_ with a hardness more than 1500 HV) balls with a diameter of 5 mm were chosen as the counterparts. The two kinds of ball counterparts, and 316 samples were used as the upper specimens and lower specimens, respectively. There were two selected conditions of dry sliding in air and grease lubrication in the friction and wear tests. Common commercial XHP lithium lubricating grease was applied under the grease lubrication conditions. A new frictional pair was prepared for each sliding test. The computer system was introduced to collect and record the friction coefficient. The tribological behaviors of the samples were defined by comparing the results of friction coefficient and mass loss. The specimens were thoroughly cleaned with acetone in an ultrasonic bath before and after each wear test. An analytical balance with an accuracy of 0.01 mg was employed to weigh the original and worn samples. The topographical features of the worn surfaces belonging to the samples were also examined using SEM and EDS [55].

## 3. Results and Discussion

The microstructural characterization of the produced 316 samples was firstly presented in this section, and the tribological behaviors were analyzed and discussed in Section 3.2 and Section 3.3.

### 3.1. Microstructural Characterizations

**Figure 3:**
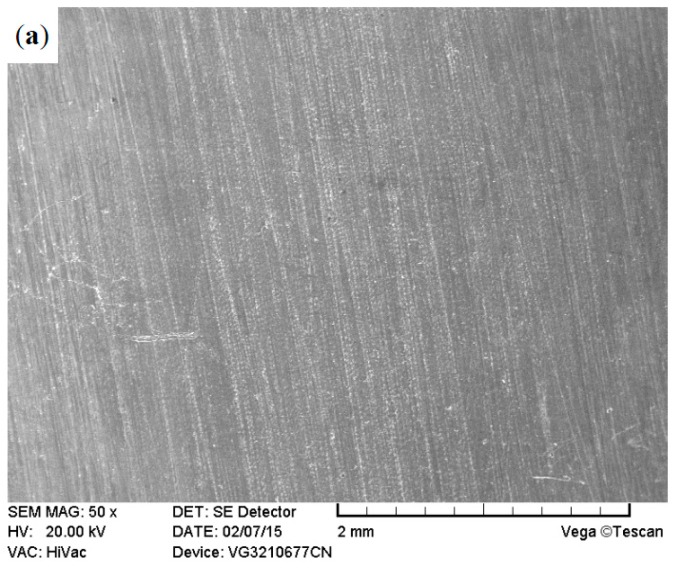

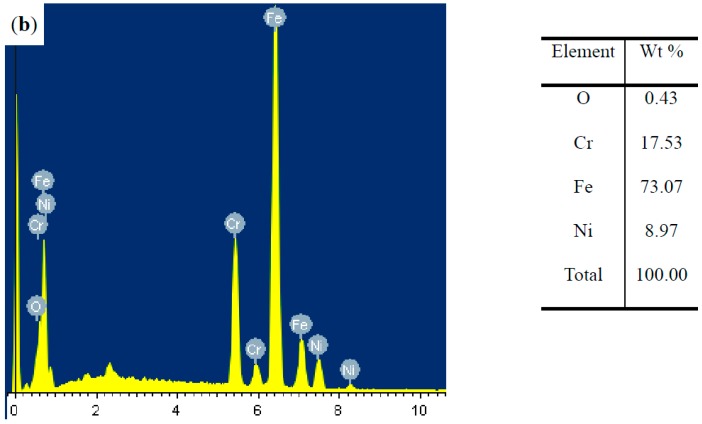
Surface morphology (**a**); and surface composition (**b**) of ground AISI 316 stainless steel.

**Figure 4:**
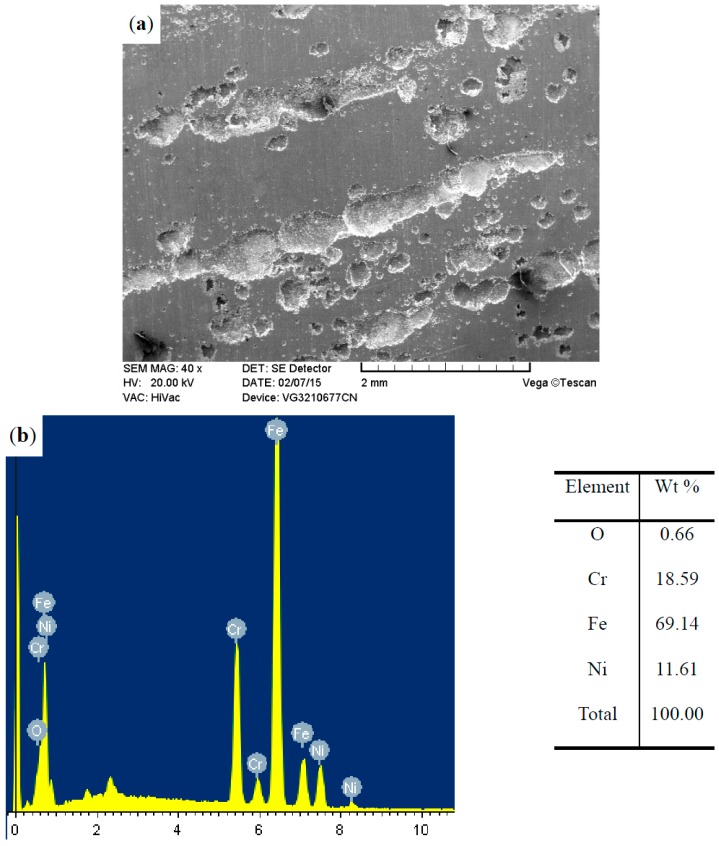
Surface morphology (**a**); and surface composition (**b**) of surface-textured 316.

**Figure 5:**
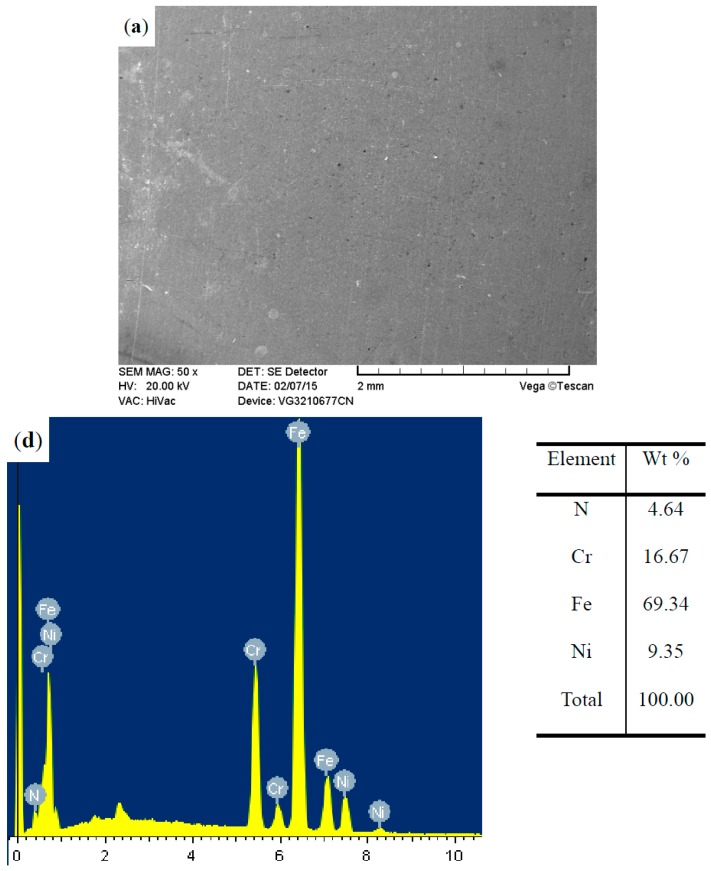
Surface morphology (**a**); and surface composition (**b**) of plasma-nitrided 316.

**Figure 6:**
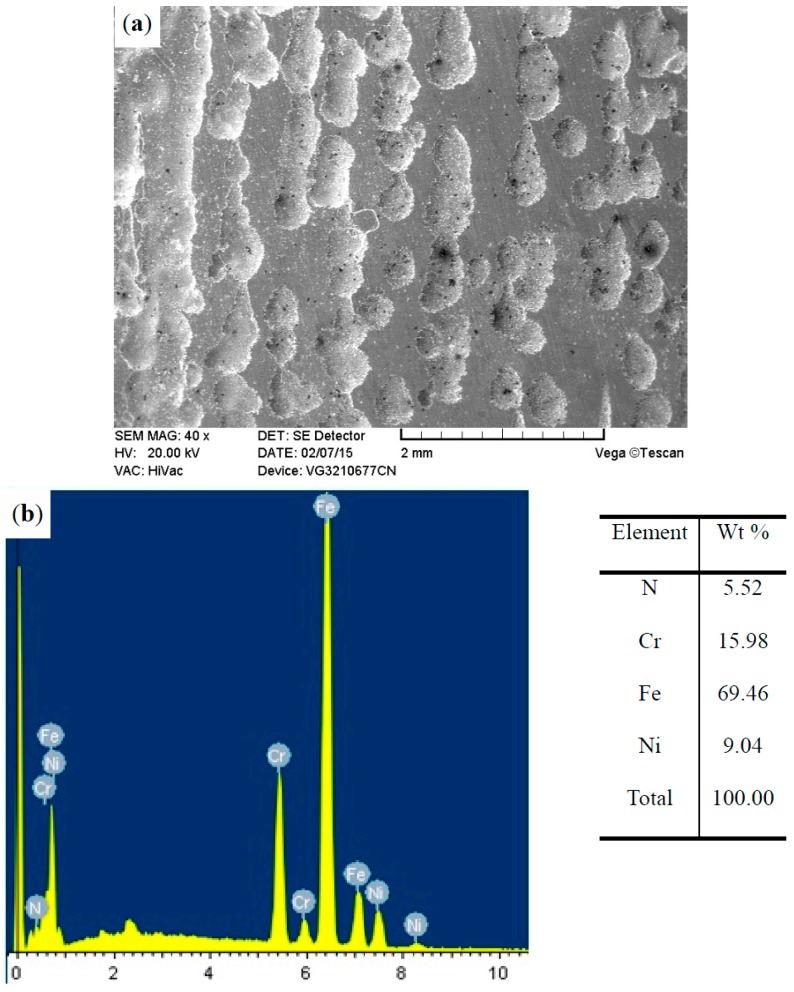
Surface morphology of duplex-treated 316 (**a**); surface composition of DT-316 (**b**).

The surface morphologies of the ground 316, ST-316, PN-316, and DT-316 were presented in Figure 3, Figure 4, Figure 5 and Figure 6. In Figure 3a, it is noticeable that numerous parallel scratches were distributed on the surface of ground 316. The scratches were formed after grinding with SiC abrasive papers. As shown in Figure 4a, the ST-316 exhibits a rougher surface than that of ground 316. Grooves and dimples which were produced after electrochemical corrosion in Cl^−^-containing solution were found on the ST-316 surface. It is well known that ASS has high pitting corrosion susceptibility in Cl^−^-rich environments, and the destructive surface of ASS‘s with numerous pits or dimples is a marked characteristic of pitting corrosion [12]. When the 316 was etched in a 15 wt % NaCl solution, the pits or dimples expanded and connected to each other, ultimately turned into pitting grooves. Figure 5a reveals that the PN-316 presented a smooth and uniform surface, most of the scratches disappeared after ion bombardment during plasma nitriding. Figure 5b presents the surface composition of PN-316, and the elemental contents are in good agreement with Li et al.’s work, which realized the formation of a nitriding layer on 316 [53].

In Figure 6, the DT-316 indicates a similar surface morphology to ST-316 and elemental contents similar to PN-316. It was found that the ion bombardment effect in plasma nitriding had a limited impact on surface morphology of DT-316, and a nitriding layer could also be successfully produced on a textured surface.

**Figure 7:**
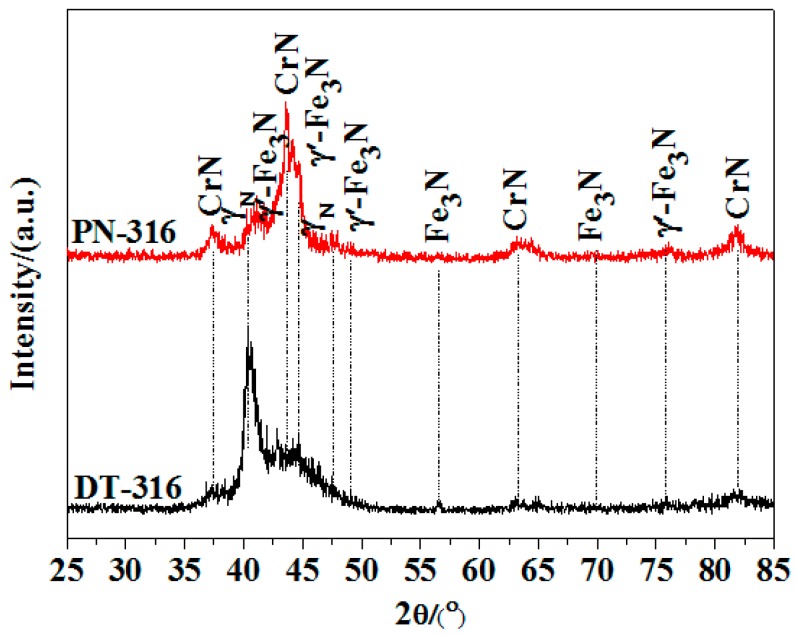
XRD patterns of PN-316 and DT-316.

Figure 7 shows the XRD patterns of PN-316 and DT-316. It was demonstrated that CrN, γ_N_, γ′-Fe_4_N, and Fe_3_N were detected in both of the PN-316 and DT-316 samples. In Devaraju and Li et al.’s studies, plasma-nitrided AISI 316L ASS samples were composed of the above phases when the plasma nitriding temperature was higher than 500 °C [17,53,54].

**Figure 8:**
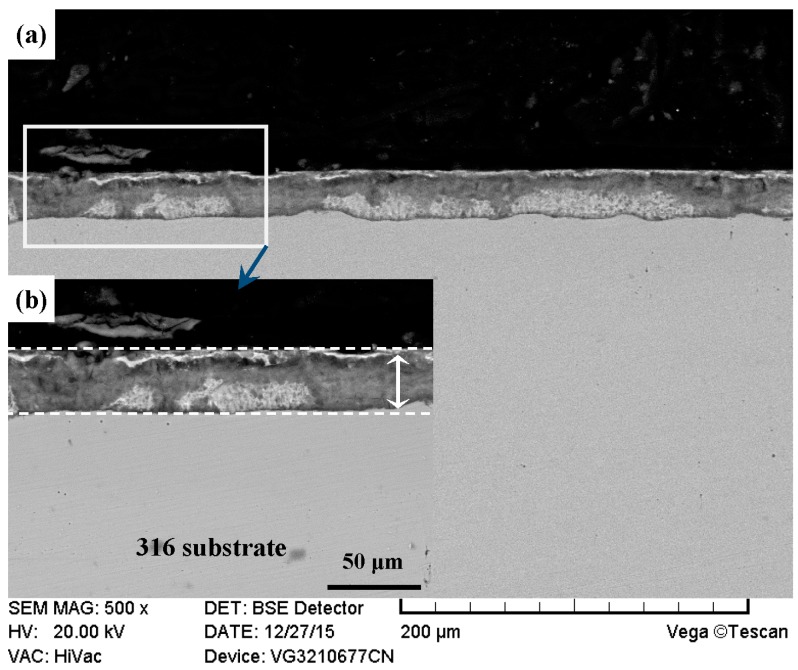
Cross sectional morphologies of the PN-316. (**a**) Low magnification; (**b**) high magnification of the white rectangle zone.

**Figure 9:**
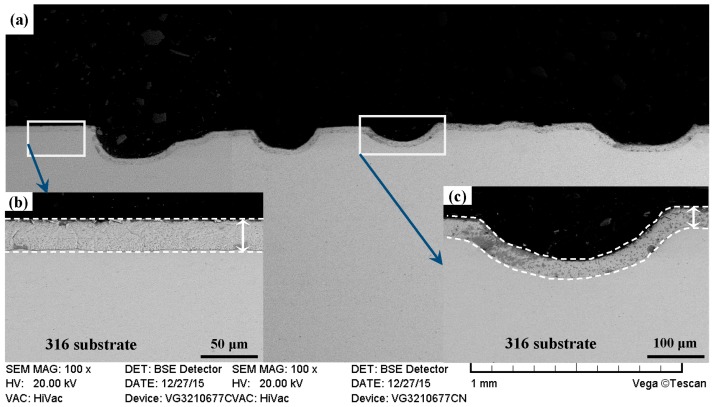
Cross sectional morphologies of the DT-316. (**a**) Low magnification; (**b**) high magnification of the white rectangle zone on left; (**c**) high magnification of the white rectangle zone on right.

Cross-sectional morphologies of PN-316 and DT-316 are suggested in Figure 8 and Figure 9. Continuous and uniform nitriding layers were formed on ground 316 and ST-316 samples, as the white dotted lines and double-headed arrows indicate. As shown in Figure 8 and Figure 9, there was no obvious difference in thickness between the two nitriding layers. Both of the two nitriding layers reached thickness values of over 30 μm. As shown in Figure 9a, a compact and continual nitriding layer was found on the surface of DT-316, which meant the DT of “surface texturing-surface plasma nitriding” was successfully realized on ground 316.

**Figure 10:**
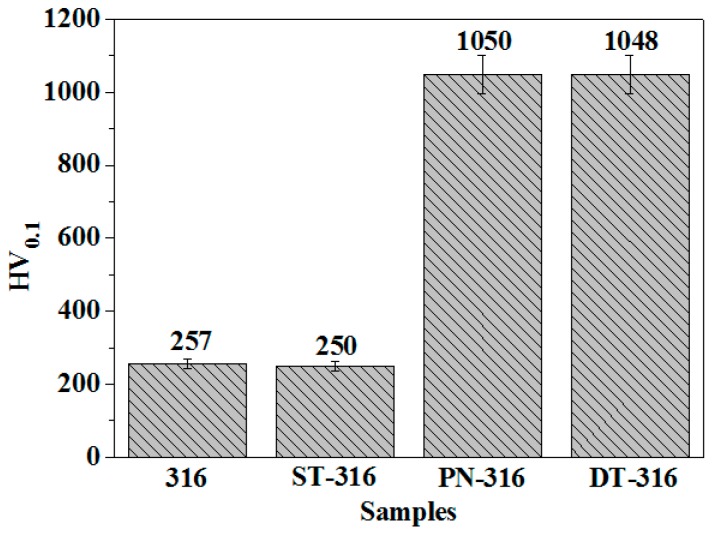
Surface hardness values of the tested samples.

A column chart of surface hardness values of ground 316, ST-316, PN-316, and DT-316 samples were created, as shown in Figure 10. It was found that the ground 316 and ST-316 samples indicated similar surface hardness values, and the hardness values of the PN-316 and DT-316 samples were similar to each other. It was seen that electrochemical treatment could form a textured surface on ground 316; however, there was no obvious change in surface hardness. As plasma nitriding was conducted on the ground 316 and ST-316 samples, the produced PN-316 and DT-316 samples showed significantly enhanced surface hardness. The improvement in surface hardness was ascribed to the formation of hard nitrides and a N-solid solution in the two nitriding layers [55].

### 3.2. Dry Sliding Tribological Behaviors

#### 3.2.1. Friction Coefficient and Mass Loss

**Figure 11:**
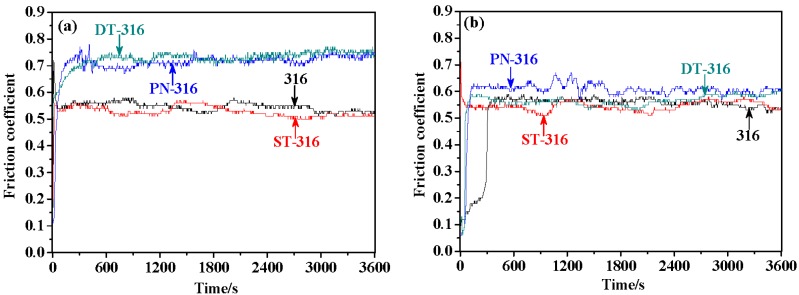
Friction coefficients of the tested samples under dry sliding—a normal load of 30 N: against GCr15 (**a**); and against Si_3_N_4_ (**b**).

Figure 11 presents the friction coefficients of the tested samples under dry sliding against GCr15 (a) and against Si_3_N_4_ (b), respectively. As shown in Figure 11a, PN-316 and DT-316 samples revealed higher friction coefficients around 0.7 than those of ground 316 and ST-316 samples, approximately 0.55. This means that there were greater friction force values between PN-316-GCr15 and DT-316-GCr15 friction pairs than ground 316-GCr15 and ST-316-GCr15 friction pairs reflected by friction coefficients. Moreover, PN-316 and DT-316 samples presented a similar variation trend and similar average values in friction coefficient, which is attributed to the formation of harder nitriding layers than GCr15 on the surfaces of related 316 samples. PN-316 and DT-316 possessing high hardness samples could resist the damage against GCr15 during dry sliding; therefore, both of them exhibited higher friction coefficients [56]. While ground 316 and ST-316 samples with lower hardness than GCr15 suffered wear damage when they slid against GCr15, they demonstrated smaller friction coefficients.

As shown in Figure 11b, all of the ground 316, ST-316, PN-316 and DT-316 samples suggested lower friction coefficients when they were sliding against Si_3_N_4_ than those friction coefficients as they were sliding against GCr15. The distinction in friction coefficient between the two counterparts could be explained as follows: The Si_3_N_4_ had far higher hardness than the above four samples. Meanwhile, the friction interfaces were ceramic–ceramic or ceramic–metal contact modes, which could reduce the tendency to adhesive wear [57]. Therefore, all four tested samples showed lower friction coefficients.

**Figure 12:**
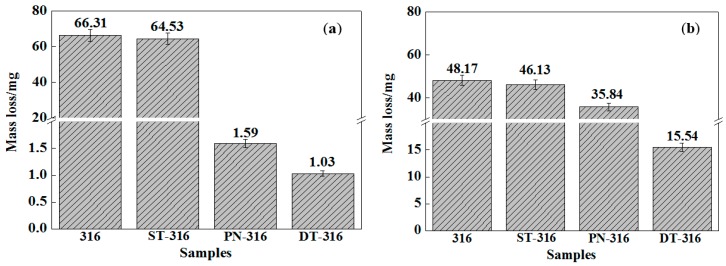
Mass losses of the tested samples under dry sliding—a normal load of 30 N: against GCr15 (**a**); and against Si_3_N_4_ (**b**).

Figure 12 compares the variation characteristics in mass losses of the tested samples under dry sliding against GCr15 (a) and against Si_3_N_4_ (b), respectively. The DT-316 presented the lowest mass loss values after sliding tests as expected (GCr15: 1.03 mg, Si_3_N_4_: 15.54 mg). The PN-316 also showed low mass losses (GCr15: 1.59 mg; Si_3_N_4_: 35.84 mg) as compared with ground 316 and ST-316 samples. It is notable in Figure 12 that ground 316 (GCr15: 66.31 mg; Si_3_N_4_: 48.17 mg) and ST-316 (GCr15: 64.53 mg; Si_3_N_4_: 46.13 mg) samples showed higher mass losses than those of PN-316 and DT-316 samples. Their mass losses were close to each other when they slid against their counterparts. This illustrated that they had undergone similar wear mechanism. However, the mass losses of PN-316 and DT-316 samples were not exactly the same, and there might be substantial differences in wear mechanism. From Figure 12, it was found that the samples with surface textures indicated lower mass losses than those samples with no surface texture. It was concluded, therefore, that surface texture played a certain role in reducing mass loss by trapping wear debris under dry sliding regardless of the counterparts in this work. Meanwhile, PN-316 and DT-316 samples with a far higher surface hardness than GCr15 were able to resist wearing damage from GCr15 counterparts regardless of dry sliding and grease lubrication.

#### 3.2.2. Wear Mechanism

**Figure 13:**
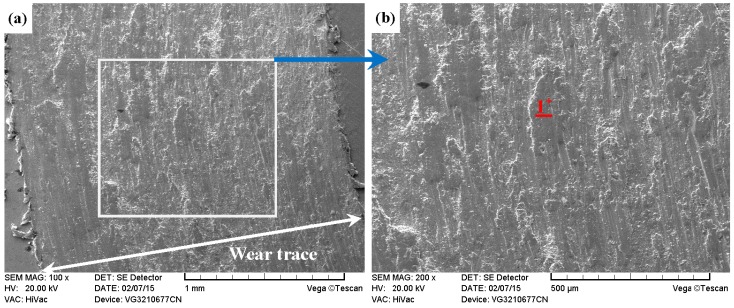
SEM images of worn surface of ground 316 after dry sliding against GCr15. (**a**) Low magnification; (**b**) high magnification of the white rectangle zone.

**Figure 14:**
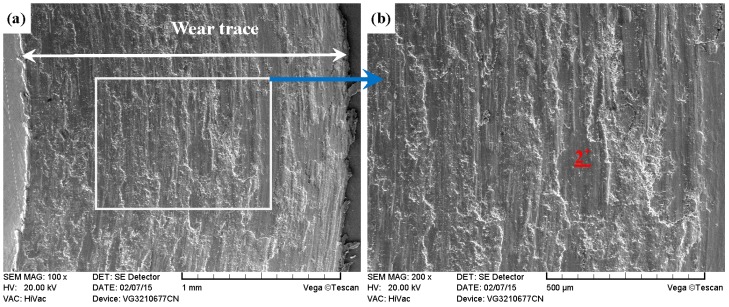
SEM images of worn surface of ST-316 after dry sliding against GCr15. (**a**) Low magnification; (**b**) high magnification of the white rectangle zone.

**Figure 15:**
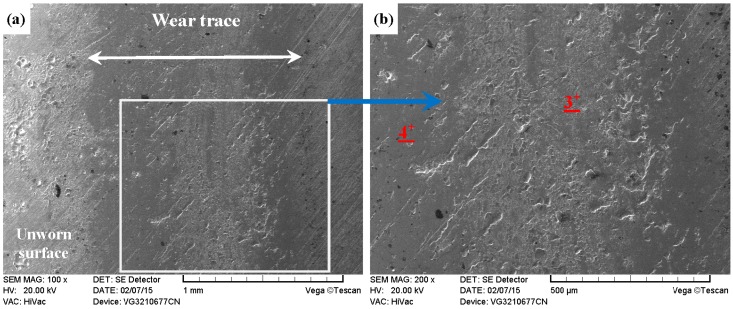
SEM images of worn surface of PN-316 after dry sliding against GCr15. (**a**) Low magnification; (**b**) high magnification of the white rectangle zone.

**Figure 16:**
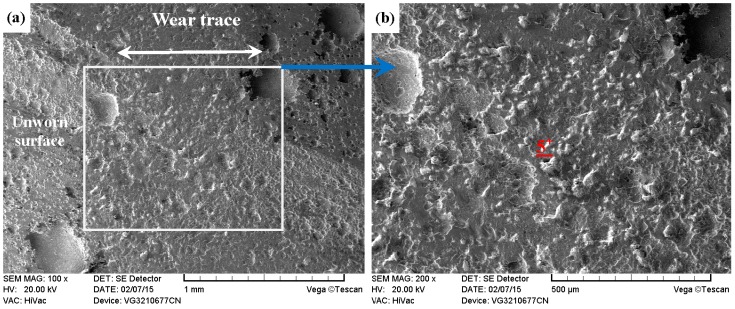
SEM images of worn surface of DT-316 after dry sliding against GCr15. (**a**) Low magnification; (**b**) high magnification of the white rectangle zone.

**Figure 17:**
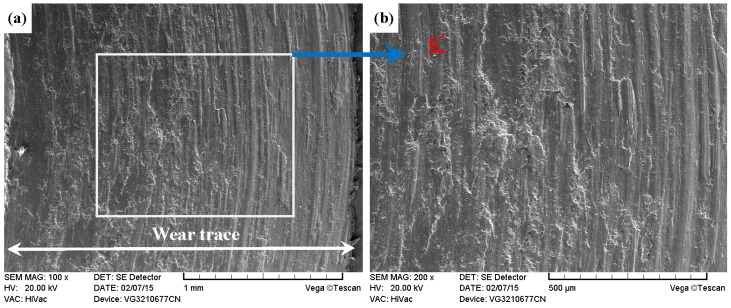
SEM images of worn surface of ground 316 after dry sliding against Si_3_N_4_. (**a**) Low magnification; (**b**) high magnification of the white rectangle zone.

**Figure 18:**
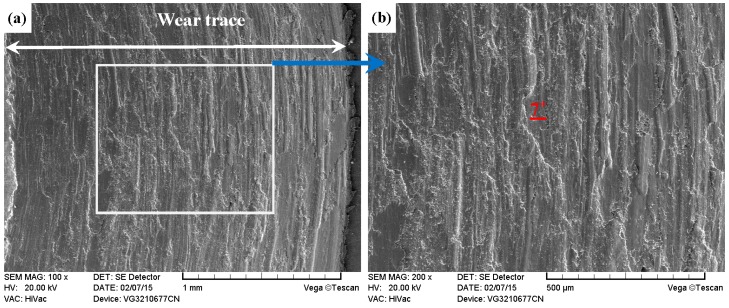
SEM images of worn surface of ST-316 after dry sliding against Si_3_N_4_. (**a**) Low magnification; (**b**) high magnification of the white rectangle zone.

**Figure 19:**
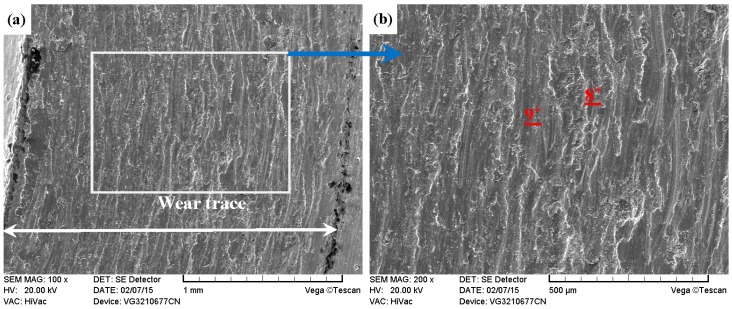
SEM images of worn surface of PN-316 after dry sliding against Si_3_N_4_. (**a**) Low magnification; (**b**) high magnification of the white rectangle zone.

**Figure 20:**
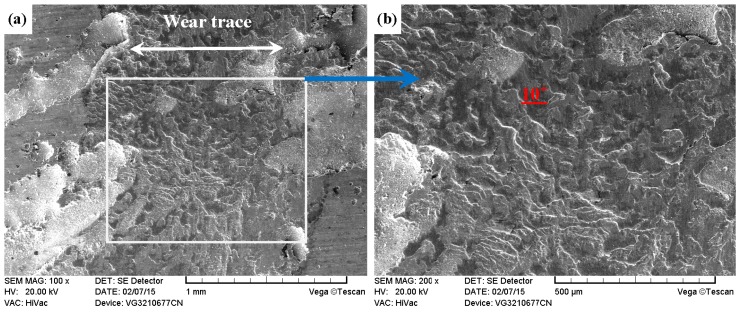
SEM images of worn surface of DT-316 after dry sliding against Si_3_N_4_. (**a**) Low magnification; (**b**) high magnification of the white rectangle zone.

In order to realize better visualizations of the worn surfaces and to explain the wear mechanisms, the worn surface morphologies of all the tested samples under dry sliding against GCr15 and against Si_3_N_4_ at different scales in the secondary electron imaging (SEI) mode obtained by SEM are given in Figure 13, Figure 14, Figure 15, Figure 16, Figure 17, Figure 18, Figure 19 and Figure 20. Meanwhile, the elemental concentrations in typical zones on the worn surfaces were characterized using EDS to provide supplementary information and then to well elucidate the deterioration mechanisms. The results are tabulated in Table 1 and Table 2.

As shown in Figure 11a, Figure 12a, Figure 13a and Figure 14a, it was found that the width values of the wear traces of the tested samples were arranged as follows: ground 316 > ST-316 > PN-316 > DT-316 when the tests were conducted under dry sliding against GCr15. In the high magnification images of the wear traces (Figure 13b, Figure 14b, Figure 15b and Figure 16b), there are obvious differences among the worn morphologies. It is notable that ground 316 and ST-316 suffered more severe wear than PN-316 and DT-316, reflecting wear traces (in Figure 13 and Figure 14) and mass losses (in Figure 13a). Meanwhile, similar wear characteristics of adhesions and abrasive scratches were found in the wear traces of ground 316 and ST-316. This can confirm that the above two samples underwent similar wearing damage and indicated a similar wear mechanism. It has been reported that, when ASS samples were sliding with themselves or other metallic materials, severe plastic deformation and adhesion junctions were frequently formed between the contacts due to the low surface hardness of ASS‘s [58]. There is also a high chemical affinity between 316 ASS and the GCr15 steel counterpart, and cold welding and adhesion junctions were prone to appear at the “metal–metal” friction interface under dry sliding. However, this kind of link with insufficient bonding strength might be broken by a relative sliding of the friction pairs, and this could result in adhesive wear. In addition, ASS was prone to oxidation during the course of dry friction in air. Table 1 presents the results of EDS analysis belonging to the samples after dry sliding against GCr15. It can be seen that both ground 316 and ST-316 underwent oxidation wear. The pull off adhesion and oxidation products were crushed to fine irregular shaped particles that could induce abrasive wear [59]. Figure 13 and Figure 14 indicate that the worn surfaces of ground 316 and ST-316 were very rough, and surface damage such as adhesive craters and abrasive scoring marks was clearly observable. Therefore, the main wear form of ground 316 and ST-316 was adhesion and abrasion, accompanied by oxidation wear [60].

As compared with Figure 13 and Figure 14, the PN-316 and DT-316 showed similar wear traces (see Figure 15 and Figure 16), which means that the PN-316 and DT-316 experienced similar wearing damage and went through a similar wear mechanism. It is certain that the plasma nitriding treated samples underwent slightly more wearing than ground 316 and ST-316 according to the width values of the wear traces (in Figure 15a and Figure 16a) and mass losses (in Figure 12a), as expected. As shown in Figure 15a and Figure 16a, the wear traces of PN-316 and DT-316 were discontinuous and incomplete. Figure 15b and Figure 16b reveal that the worn surfaces of PN-316 and DT-316 were not as rough as the worn surfaces of ground 316 and ST-316. Meanwhile, Figure 12a indicates that the mass losses of PN-316 and DT-316 were far lower than those of ground 316 and ST-316. The promising wear resistance of PN-316 and DT-316 is attributed to the changing of contact mode from “metal–metal” to “ceramic–metal” and the increased surface hardness (formation of nitride) after plasma nitriding [58]. Figure 15b and EDS analysis in Table 1 show that the local area on the worn surface of PN-316 was destroyed. It is certain that DT-316 was not worn through after dry sliding against GCr15, which was also reflected by EDS analysis. Moreover, GCr15 was softer than PN-316 and DT-316; hence, GCr15 balls were more likely to be destroyed when they were sliding against PN-316 and DT-316. The main wear mechanism of PN-316 and DT-316 was mild abrasive wear and transfer from GCr15 to their surfaces in accordance with the observations of worn surfaces.

As shown in Figure 17a, Figure 18a, Figure 19a and Figure 20a, it was clear that the width of the tested samples can also be arranged in the following sequence: ground 316 > ST-316 > PN-316 > DT-316 when the tests were processed under dry sliding against Si_3_N_4_. As shown in the higher magnification images of the wear traces (Figure 17b, Figure 18b, Figure 19b and Figure 20b), there were remarkable differences among the worn morphologies. As presented in Figure 17b and Figure 18b, numerous parallel deep grooves and spalled holes were found on the wear surfaces of ground 316 and ST-316. Micro-protuberances on Si_3_N_4_ played a ploughing effect on the soft ASS surface and left ploughing grooves on the wear surfaces. As the Si_3_N_4_ ball was far harder than ASS, plastic deformation took place on the ASS under the actions of load and friction force. There was a dislocation pileup region on the near surface, which worked to induce the initiation of micro-cracks underneath the surface [61]. The cracks gradually propagated with the development of plastic deformation as the cracks extended to the surface. As a result, thin and long wear sheets were formed and removed from the surface. The wear sheets were ground into abrasive particles of a small size by the relative motion of friction pair, and abrasive wear then occurred [61]. Thereby, the wear mechanism of ground 316 and ST-316 was a composite form of delamination and abrasive wear.

According to mass loss results in Figure 12b and EDS analysis in Table 2, PN-316 was worn through after dry sliding against Si_3_N_4_. PN-316 suffered heavy wear, and it was evident that scratches and ploughing grooves were found on the worn surface (see Figure 19). The main wear manner of PN-316 under this condition was set as abrasive wear [58].

Figure 12b shows that DT-316 presented the lowest mass loss, and Figure 20a demonstrates that DT-316 also had the narrowest wear trace. As shown in Figure 20b, cracking and spalling were found on the worn surface. The DT-316 had a high hardness value and textured surface, which caused high contact stress in the friction interface [62]. DT-316 was subjected to cyclic loading during the sliding, and the cyclic loading had a very significant effect in initiation and growth of fatigue crack. Once the crack propagation ran to a degree, spalling off occurred on the worn surface. Si_3_N_4_ possesses higher hardness and higher chemical stability, and DT-316 was going to be worn. Therefore, the main wear mechanism of DT-316 was fatigue wear.

Combining the mass losses with the observations of wearing morphologies, it was confirmed that the DT-316 exhibited the best wear resistance under dry sliding against GCr15 and Si_3_N_4_ as compared with ground 316, ST-316, and PN-316.

### 3.3. Sliding Tribological Behaviors under Grease Lubrication

#### 3.3.1. Friction Coefficient and Mass Loss

**Figure 21:**
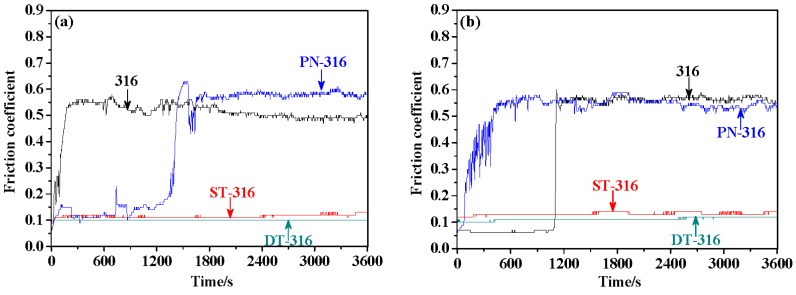
Friction coefficients of the tested samples under grease lubrication—a normal load of 30 N: against GCr15 (**a**); and against Si_3_N_4_ (**b**).

Figure 21 shows the friction coefficients of the tested samples under grease lubrication against GCr15 (a) and against Si_3_N_4_ (b), respectively. As shown in Figure 21a, PN-316 and ground 316 samples revealed low friction coefficients in the running-in stage, a layer of grease and a thin sorption film on their surfaces contributed to the reduction of friction. PN-316 and ground 316 samples had a relative smooth surface and could not store up grease. As the grease was squeezed out of the friction interface and the thin sorption film was worn down, the contact mode turned into nearly dry sliding. Increased friction coefficients belonging to PN-316 and ground 316 fluctuated in the range of 0.5~0.6, which were higher than those of ST-316 and DT-316 samples around 0.1. As ST-316 and DT-316 had textured surfaces, the textured surfaces could play a role of storing up grease. The grease that was stored in the grooves and dimples provided continuous lubrication, so they exhibited far lower friction coefficients in the entire testing period. As shown in Figure 21b, PN-316 and ground 316 also showed similar friction coefficient values as given in Figure 21a. ST-316 and DT-316 with textured surfaces indicated a far lower friction coefficient as well.

**Figure 22:**
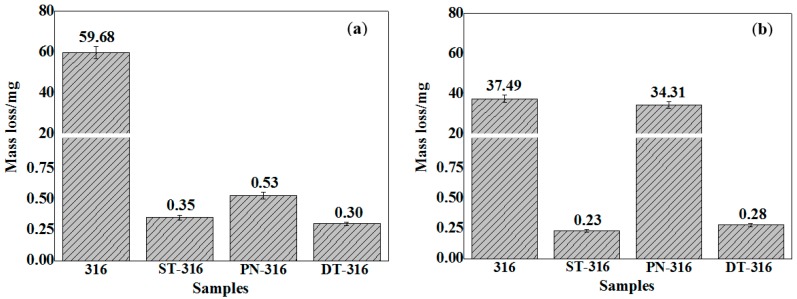
Mass losses of the tested samples under grease lubrication—a normal load of 30 N: against GCr15 (**a**); and against Si_3_N_4_ (**b**).

As shown in Figure 22, ground 316 presented the highest mass losses of all the tested samples under grease lubrication against GCr15 (a) and against Si_3_N_4_ (b), and ST-316 and DT-316 had much fewer mass losses than ground 316. PN-316, which underwent different wear modes, presented low mass loss after sliding against GCr15 and relatively high mass loss after sliding against Si_3_N_4_. Combining Figure 22 with the friction coefficient results in Figure 21, it can be seen that PN-316 experienced slight wear after sliding against GCr15, and it was not worn down. However, PN-316 received heavy wear from Si_3_N_4_, and it was worn through, reflected by the friction coefficient and mass loss, as compared with ground 316.

#### 3.3.2. Wear Mechanism

**Figure 23:**
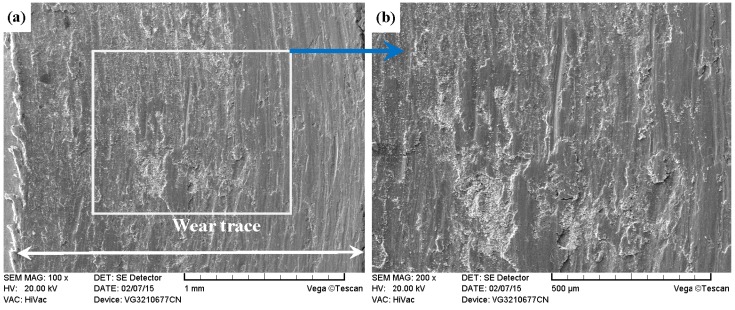
SEM images of worn surface of ground 316 under grease lubrication against GCr15. (**a**) Low magnification; (**b**) high magnification of the white rectangle zone.

**Figure 24:**
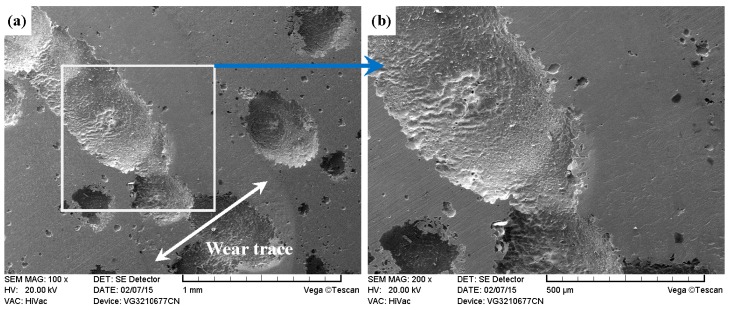
SEM images of worn surface of ST-316 under grease lubrication against GCr15. (**a**) Low magnification; (**b**) high magnification of the white rectangle zone.

Figure 23a shows that the width value of the wear trace belonging to ground 316 under grease lubrication against GCr15 was close to the tested ground 316 sample after dry sliding against GCr15. It was also illustrated that the ground 316 received similar damages under the two conditions by mass losses (in Figure 12a and Figure 22a). The high magnification image of the wear trace in Figure 23b showed a worn morphology similar to Figure 13b, indicating that ground 316 had a similar wear mechanism of adhesive wear and abrasive wear. Figure 24a indicates that the wear trace of ST-316 was incomplete and discontinuous, groove and dimples were found in the wear trace. Figure 24b shows that there were no ploughing or scratching features on the worn surface. The worn region was smooth, like a polished surface. As established in Figure 21a, Figure 22a and Figure 24b, the ST-316 with a textured surface was been destroyed. Grooves and dimples on the textured surface acted as a grease reservoir to realize the friction reduction [62,63]. The wear mechanism of ST-316 can be deduced as a polishing-like degradation.

**Figure 25:**
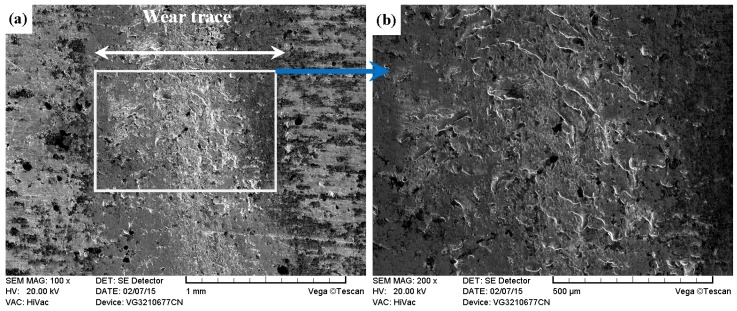
SEM images of worn surface of PN-316 under grease lubrication against GCr15. (**a**) Low magnification; (**b**) high magnification of the white rectangle zone.

When PN-316 was tested under grease lubrication against GCr15, its superior wear resistance compared with that of ground 316 benefitted from its higher surface hardness. Figure 25a reveals a narrower wear trace than that of ground 316 in Figure 23a. Grease lubrication in the running-in stage and a contact mode of “ceramic–metal” contributed to a lower mass loss and slight wear of PN-316 under grease lubrication against GCr15 compared with those after dry sliding against GCr15. The wear mechanism of PN-316 still showed slight abrasive wear and transfer from GCr15 to its surface (see Figure 25b).

**Figure 26:**
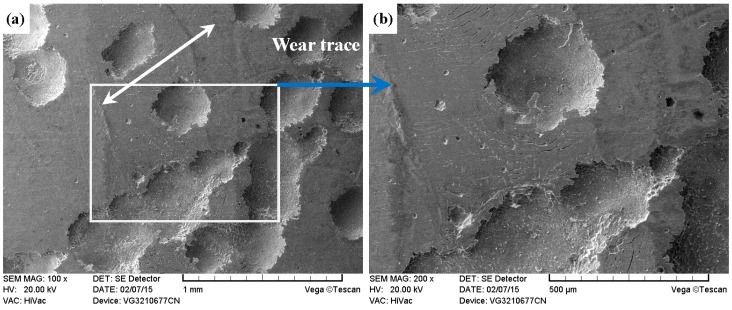
SEM images of worn surface of DT-316 under grease lubrication against GCr15. (**a**) Low magnification; (**b**) high magnification of the white rectangle zone.

As shown in Figure 26a, it was found that the wear trace of DT-316 under grease lubrication against GCr15 was not clear. No obvious ploughing or scratching can be found in the high magnification image of the wear trace in Figure 26b. As shown in Figure 26b, the worn zone on the DT-316 surface is also smoother than that of PN-316 in Figure 25b. However, cracks that were perpendicular to the sliding direction can be observed in Figure 25b, which meant mild fatigue wear had occurred.

**Figure 27:**
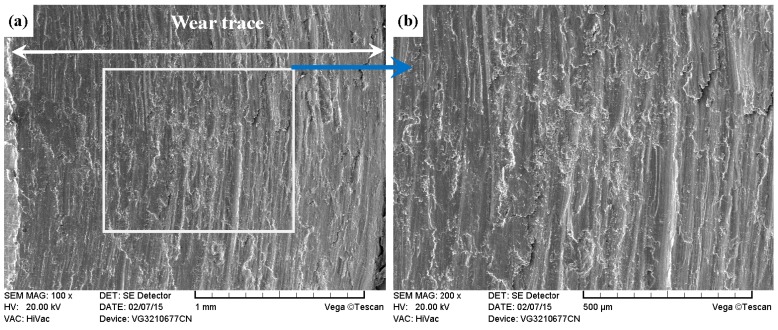
SEM images of worn surface of ground 316 under grease lubrication against Si_3_N_4_. (**a**) Low magnification; (**b**) high magnification of the white rectangle zone.

As the ground 316 could not store grease and the grease would be squeezed out of the friction interface, the friction pairs directly contacted each other. As a result, ground 316 was severely worn by Si_3_N_4_ in spite of the test that had begun with grease lubrication. Figure 27 suggests a wear morphology similar to Figure 17 and confirms that ground 316 also underwent delamination and abrasive wear under grease lubrication.

**Figure 28:**
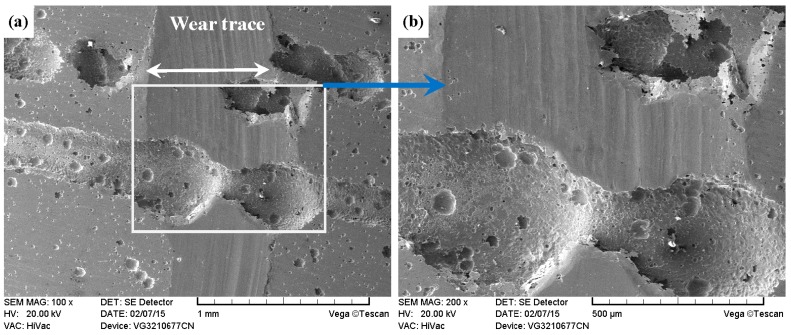
SEM images of worn surface of ST-316 under grease lubrication against Si_3_N_4_. (**a**) Low magnification; (**b**) high magnification of the white rectangle zone.

As shown in Figure 28a, the ST-316 obtained a wear trace with an inconformity in width, and shallow grooves are found in Figure 28b. Because Si_3_N_4_ was far harder than ST-316, plastic deformation would occur under the given normal load during sliding. Some of the stored grease on textured surface were squeezed out and participated in the friction process at the friction interface. The stored grease on textured surface could provide continuous lubrication, and played positive effects on friction reduction and wear reducing. Thereby, the wear mechanism of ST-316 was mild abrasive wear.

**Figure 29:**
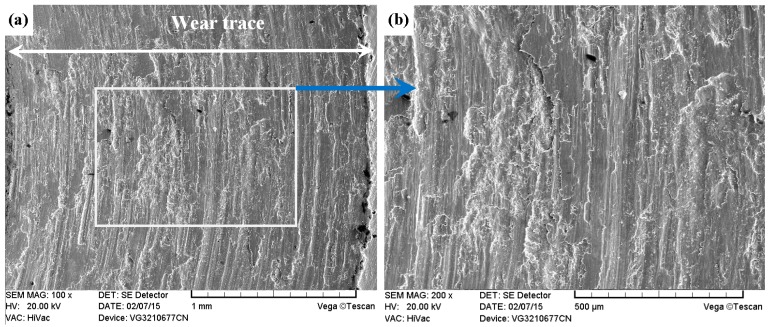
SEM images of worn surface of PN-316 under grease lubrication against Si_3_N_4_. (**a**) Low magnification; (**b**) high magnification of the white rectangle zone.

According to the friction coefficient and mass loss in Figure 21b and Figure 22b, it is certain that PN-316 was worn through under grease lubrication against Si_3_N_4_. As shown in Figure 29a, PN-316 with a wide wear trace experienced severe wear and the worn surface was very rough. As shown in Figure 29b, it is obvious that scratches and ploughing grooves were distributed on the worn surface. The wear manner of PN-316 under grease lubrication against Si_3_N_4_ was abrasive wear.

**Figure 30:**
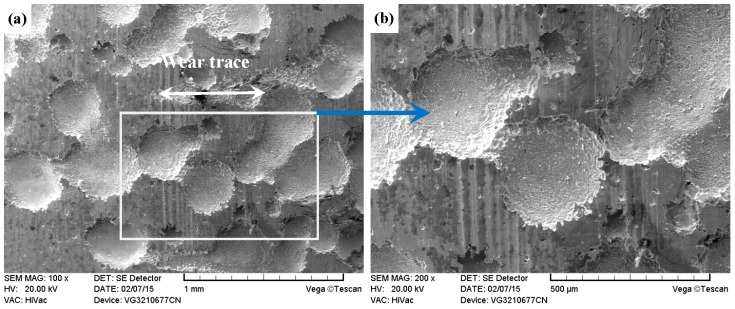
SEM images of worn surface of DT-316 under grease lubrication against Si_3_N_4_. (**a**) Low magnification; (**b**) high magnification of the white rectangle zone.

As shown in Figure 30a, the wear trace of DT-316 under grease lubrication against Si_3_N_4_ was illegible, and pits and dimples were found inside and outside the worn zone. Combined with the friction coefficient and mass loss in Figure 21b and Figure 22b, it was confirmed that DT-316 received slight wearing damage. Parallel shallow grooves and minor cracks were found in the wear trace, as indicated in Figure 30b. The textured surface distributed with grooves, dimples, or pits could store up grease and provided continuous lubrication during friction, which was good for friction reduction and wear reducing [62]. However, Si_3_N_4_ had higher hardness, and its micro-protuberances on the surface could bring damages such as ploughing or scratching to DT-316. In addition, there was a nitriding layer which had high hardness but low plasticity on DT-316, it was difficult to get plastic deformation for the nitriding layer. Meanwhile, the nitriding layer could not fully resist the wearing from Si_3_N_4_, fatigue cracks appeared in the local worn area. Therefore, the main wear mechanism of DT-316 was abrasive wear accompanied by slight fatigue wear.

## 4. Conclusions

**Figure A1:**
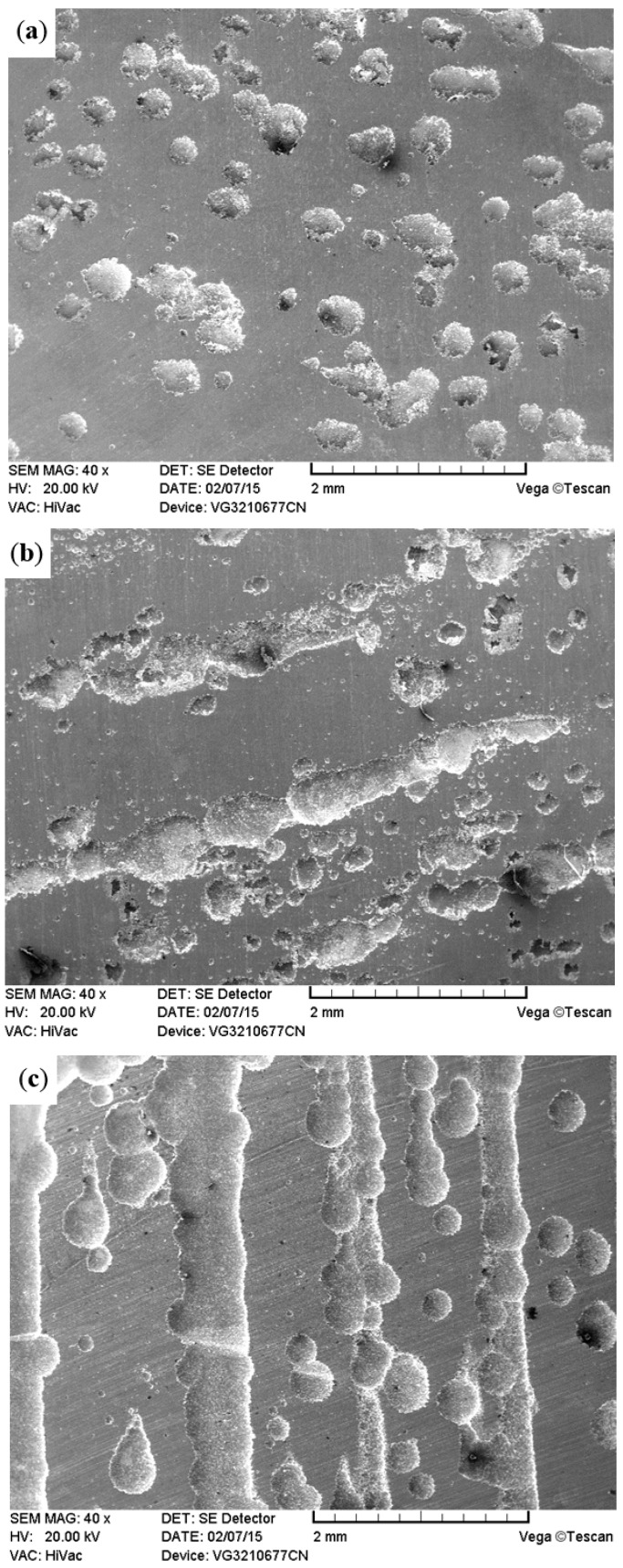

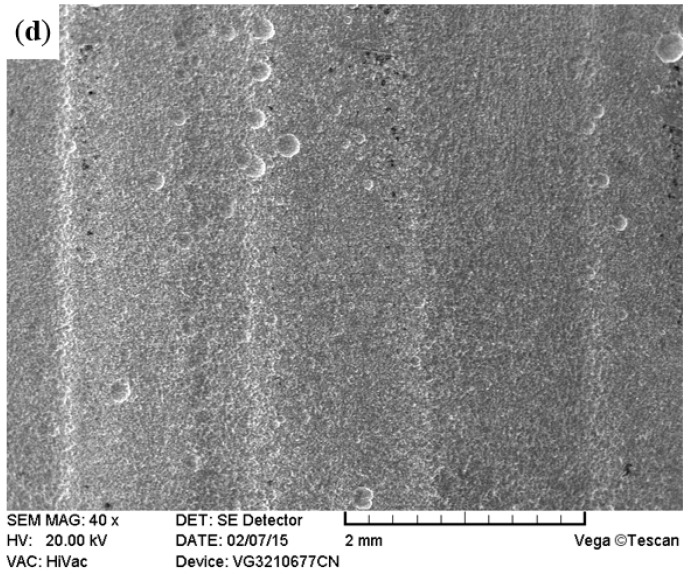
Surface morphologies of the electrochemical processed 316 surfaces. (**a**) 10 wt % NaCl; (**b**) 15 wt % NaCl; (**c**) 20 wt % NaCl; (**d**) 25 wt % NaCl.

Duplex treatment of surface texture-plasma nitriding was conducted in virtue of the advantages of its surface texture and surface modification. Firstly, the 316 substrate was electrochemically processed in a NaCl solution to obtain a textured surface (ST-316) by making use of its high sensitivity to pitting corrosion in a Cl^−^-rich environment. Then, the plasma nitriding was employed to treat the 316 substrate and ST-316. The tribological behaviors of ground 316, ST-316, PN-316, and DT-316 were thoroughly investigated, and the findings were summarized as follows:

(1) When NaCl media with high concentrations were used to achieve electrochemical processing of 316 samples on purpose, the initial formed pits expanded and attached to each other, ultimately turning into grooves and dimples that distributed on the surfaces (See Figure A1).

(2) A continuous and uniform nitriding layer was successfully prepared on ground 316 and ST-316 surfaces. The obtained nitriding layers reached thickness values of over 30 μm. The nitriding layer was built up by iron nitrides and chromium nitride. Plasma nitriding significantly enhanced the surface hardness of the 316 substrate and ST-316 samples.

(3) In dry sliding against GCr15, ground 316 and ST-316 with lower surface hardness suffered more severe wear than PN-316 and DT-316. Ground 316 and ST-316 underwent adhesive wear and abrasive wear, accompanied with oxidation wear. ST-316 revealed tribological behavior similar to ground 316, which meant single surface texturing presented no obvious positive effect on the tribological performance of ground 316 in dry sliding against GCr15. Plasma nitriding treated samples underwent slighter wearing than ground 316 and ST-316, as expected. The mass losses of PN-316 and DT-316 were far lower than those of ground 316 and ST-316. The main wear mechanism of PN-316 and DT-316 was mild abrasive wear and transfer from GCr15 to their surfaces.

(4) In dry sliding against Si_3_N_4_, there were remarkable differences among the worn morphologies. The wear mechanism of ground 316 and ST-316 was a composite form of delamination and abrasive wear. The main wear manner of PN-316 under this condition was set as abrasive wear. DT-316 experienced fatigue wear.

(5) Under grease sliding against GCr15, ground 316 received a similar extent of damages as it was in dry sliding, it revealed a wear mechanism of adhesive wear and abrasive wear. The ST-316 with a textured surface was not destroyed. Grease stored in grooves and dimples on the textured surface could realize the friction reduction. The wear mechanism of ST-316 can be deduced as polishing-like degradation. The wear mechanism of PN-316 was slight abrasive wear and transfer from GCr15 to its surface. No obvious ploughing or scratching was found in the wear trace of DT-316. Minor cracks were observed, which meant DT-316 received mild fatigue wear.

(6) Under grease sliding against Si_3_N_4_, although the test began with grease lubrication, ground 316 was still severely worn. Ground 316 also underwent delamination and abrasive wear. The wear mechanism of ST-316 was mild abrasive wear. PN-316 was worn through under this condition, and its wear manner was abrasive wear. DT-316 received slight wearing damage. Ploughing, scratching, and fatigue cracks appeared in a local worn area on the worn surface of DT-316. The main wear mechanism of DT-316 was abrasive wear accompanied by slight fatigue wear.

(7) When the tribological tests were carried out under dry sliding and grease lubrication conditions, the tested samples displayed different tribological behaviors. The PN-316 indicated a secondary improvement in wear resistance compared with the DT-316, as they were estimated under dry sliding, while the tribological properties of ST-316 could be ranked only second to those of the DT-316 when the tests were conducted under grease lubrication conditions.

(8) The DT-316 samples revealed the most promising tribological properties, reflecting the lowest mass loss and worn morphologies. The excellent tribological performance of DT-316 was attributed to the following aspects: firstly, the nitriding layer had a high surface hardness; secondly, the surface texture was able to capture wear debris, store up grease, and then provide continuous lubrication. Therefore, surface texturing-plasma nitriding duplex treatment could be performed on the working surface of 316 stainless steel with enhanced surface hardness and good tribological properties. The surface texturing-plasma nitriding surface treatment duplex processing realized a “1 + 1 > 2” effect on 316 SS. Our group will concentrate on choosing appropriate surface technologies on textured surface for specific serving conditions. Meanwhile, tribo-chemical reactions between the counterparts and the grease, or between the tested samples and the grease, are also worthy of attention.

## Figures and Tables

**Table 1:** EDS analysis of selected zones on worn surfaces after dry sliding against GCr15.

Element (wt %)	O	N	Fe	Cr	Ni
Zone-1	11.18	–	63.53	16.05	9.24
Zone-2	11.14	–	63.24	16.48	9.14
Zone-3	2.21	3.65	68.35	16.41	9.38
Zone-4	–	4.64	69.34	16.67	9.35
Zone-5	26.26	0.11	70.94	2.25	0.44

**Table 2:** EDS analysis of selected zones on worn surfaces after dry sliding against Si_3_N_4_.

Element (wt %)	O	N	Si	Fe	Cr	Ni
Zone-6	11.75	0.04	0.72	61.52	15.98	9.99
Zone-7	12.64	0.05	0.62	61.21	15.94	9.54
Zone-8	12.90	0.49	0.46	61.61	15.58	8.96
Zone-9	1.23	0.04	0.29	70.10	18.20	10.14
Zone-10	1.16	1.14	0.35	69.09	17.75	10.51

## Appendix A

Figure A1 shows the SEM surface morphologies of the electrochemical processing 316 samples in different NaCl concentrations. As can be seen from Figure A1, all processed surfaces had become rougher compared with the ground 316 (Figure 3a). Electrochemical processing of 316 samples in a 10 wt % NaCl solution yields a surface with numerous randomly distributed corrosion pits (Figure A1a). These data are consistent with published works, where little difference was observed in the surface morphologies of pitting corrosion of 316. When the 316 was processed in a 15 wt % NaCl solution, the pits expand and become attached to each other, ultimately turned into pitting grooves and dimples, as shown in Figure A1b (and Figure 1b in this paper). In a 20 wt % NaCl solution, the surface seems to contain more parallel distributed pitting grooves and dimples (Figure A1c), indicating a certain surface texture characteristic. On the other hand, increasing the concentration of NaCl was not likely going to produce better surface texture characteristics. As demonstrated in Figure A1d, a relatively uniform and smooth surface was obtained after the electrochemical etching process in a 25 wt % NaCl solution. As a result, we concur with the conclusion that higher concentrations of Cl^−^ combined with relatively elevated scan rates might yield severe general corrosion of the 316 surface, indicated by wide and shallow grooves, and tiny pits.
